# Robotic versus open oncological gastric surgery in the elderly: a propensity score-matched analysis

**DOI:** 10.1007/s11701-020-01168-2

**Published:** 2020-11-05

**Authors:** Giovanni Maria Garbarino, Gianluca Costa, Barbara Frezza, Alessia Biancafarina, Genoveffa Balducci, Paolo Mercantini, Marco De Prizio, Giovanni Gugliemo Laracca, Graziano Ceccarelli

**Affiliations:** 1 Department of Medical Surgical Sciences and Translational Medicine, Sapienza University of Rome, Sant’Andrea Hospital, Via di Grottarossa 1035-39, 00189 Rome, Italy; 2 Division of General Surgery, Department of Surgery, San Donato Hospital, via Pietro Nenni 20-22, 52100 Arezzo, Italy; 3 Division of General Surgery, Department of Surgery, San Giovanni Battista Hospital, Local Health Service Umbria 2, via Massimo Arcamone 1, 06034 Foligno, PG Italy

**Keywords:** Gastric cancer, Robotic surgery, Minimally Invasive Surgery, Gastric Surgery

## Abstract

Although there is no agreement on a definition of elderly, commonly an age cutoff of ≥ 65 or 75 years is used. Even if robot-assisted surgery is a validated option for the elderly population, there are no specific indications for its application in the surgical treatment of gastric cancer. The aim of this study is to evaluate the safety and feasibility of robot-assisted gastrectomy and to compare the short and long-term outcomes of robot-assisted (RG) versus open gastrectomy (OG). Patients aged ≥ 70 years old undergoing surgery for gastric cancer at the Department of Surgery of San Donato Hospital in Arezzo, between September 2012 and March 2017 were enrolled. A 1:1 propensity score matching was performed according to the following variables: age, Sex, BMI, ASA score, comorbidity, T stage and type of resection performed. 43 OG were matched to 43 RG. The mean operative time was significantly longer in the RG group (273.8 vs. 193.5 min, *p* < 0.01). No differences were observed in terms of intraoperative blood loss, an average number of lymph nodes removed, mean hospital stay, morbidity and mortality. OG had higher rate of major complications (6.9 vs. 16.3%, OR 2.592, 95% CI 0.623–10.785, *p* = 0.313) and a significantly higher postoperative pain (0.95 vs. 1.24, *p* = 0.042). Overall survival (*p* = 0.263) and disease-free survival (*p* = 0.474) were comparable between groups. Robotic-assisted surgery for oncological gastrectomy in elderly patients is safe and effective showing non-inferiority comparing to the open technique in terms of perioperative outcomes and overall 5-year survival.

## Introduction

Gastric cancer (GC) is the fifth most common cancer and the third leading cause of cancer-related death worldwide [1].

Population aging continues to grow with significant implications for each national health system, particularly in western countries, increasing the burden of resources for assistance [2, 3]. With this trend, cancer will become a disease of the elderly. In this aged population, cancer often presents itself as an advanced stage of the disease. Therefore, the overall 5-year survival rate of patients with gastric cancer in western countries is around 25% [4].

Elderly patients compared to younger ones often have one or more comorbidities and are often "fragile"; are at greater risk of morbidity and mortality. The frail elderly are less able to tolerate the stress of medical diseases, hospitalizations and immobility; as a result, surgery can be a substantial problem in this population, showing an increase of complication rates, mortality, length of hospital stay and ICU admissions [5].

In this sense, laparoscopic surgery has been shown to be better tolerated than open surgery in the selected elderly population [6].

Robot-assisted surgery allows multiple patients to benefit from minimally invasive surgery (MIS), overcoming many laparoscopic drawbacks and limitations [7]. It is used and widely accepted in general surgery and in particular in oncological surgery [8–11]. Concerning oncological gastric surgery, the robot-assisted technique has been shown to have many advantages compared to open technique, such as less blood loss, early bowel movements, faster mobilization, and shorter hospital stay [9, 12–14]. Despite that, there are no specific guidelines for its application in gastric cancer patients.

The aim of this study is to evaluate the safety and feasibility of robotic gastrectomy in the elderly comparing the short and long-term outcomes of robotic (RG) versus open gastrectomy (OG).

## Patients and methods

This is a retrospective observational study concerning all consecutive patients undergoing surgery for gastric cancer at the Department of Surgery of San Donato Hospital in Arezzo between September 2012 and March 2017. Data were retrieved from a prospectively maintained database including all patients undergoing any gastric procedure for both benign and malignant condition. The research was undertaken as a part of a residency program of the Department of Medical Surgical Sciences and Translational Medicine of Sapienza University of Rome in Sant’Andrea Teaching Hospital. Data included in this study were: demographics (age, sex, ASA score, BMI, comorbidities), tumour characteristics, operative details, tumour pathology, short-term outcomes, overall survival. The following inclusion criteria were considered: (1) patients undergoing minimally invasive or open gastrectomy for a histologically proven gastric carcinoma with pre-operative CT staging and multidisciplinary team evaluation; (2) patients ≥ 70 years old; (3) at least D2 lymphadenectomy, and (4) procedure performed by the senior surgeon. The exclusion criteria were: (1) patients < 70 years old; (2) patients with tumour located at the gastro-oesophageal junction.

Patients were divided into two groups according to the surgical approach: robotic (RG group) and open (OG group).

Robotic procedures converted to open were analysed according to the intention-to-treat principle and included in the RG group.

The primary endpoint was to compare the short-term outcomes between open and robotic approach focusing on morbidity and mortality within 30 days from surgery. As secondary endpoints, long-term 5-year overall survival (OS) and disease-free survival (DFS) were evaluated and compared between groups.

All patients were discussed in a multidisciplinary environment involving surgeons, medical oncologists, radiologists and pathologists.

This study was conducted in accordance with the Declaration of Helsinki and its later amendments or comparable ethical standards. A formal Institutional Review Board approval was not required because of the non-interventional retrospective design; however, a signed consent for the treatment and the analysis of data for the scientific purpose was obtained from all patients before any surgical procedures.

### Definitions

Comorbidities were categorized according to the Charlson Age Comorbidities Index (CACI).

Preoperative clinical staging was assessed in all patients by total body CT scan and endoscopic ultrasound when indicated.

Tumour staging was performed according to 7th TNM edition [15].

A 4-point Verbal Rating Scale (VRS) scale was used for the evaluation of postoperative pain: 0 point corresponds to the absence of pain, one point to some pain, two points to considerable pain and three points to a pain which could not be more severe.

Morbidity and mortality were defined as postoperative complications and death within 30 days from surgery respectively. Morbidity was graded according to the Clavien-Dindo classification and complications graded ≥ III were defined as major [16].

Reoperation was defined as every surgical procedure following primary surgery during hospitalization or within 30 days after a primary intervention.

Overall survival (OS) was defined as the time between surgery and death for any cause or last follow-up.

No patients underwent neoadjuvant chemotherapy and patients were treated with adjuvant chemotherapy (oxaliplatin and capecitabine) after surgery when indicated as proposed in the “Associazione Italiana di Oncologia Medica” (AIOM) guidelines at the time of the surgical treatment [17].

### Statistical analysis

Continuous data were expressed as the mean (± standard deviation) or median and interquartile range (IQR 25–75%) depending on their distribution that was assessed through the Shapiro–Wilks test. Unpaired Student *t* test was used to compare differences in continuous parametric variables and the Mann–Whitney *U* test for continuous nonparametric variables. Numbers and percentages were used for reporting categorical variables and the *χ*^2^ test or Fisher’s exact test with or without Yates correction were used for comparisons.

A propensity score matching was applied to eliminate selection bias between groups and was reported according to the recommendations of Lonjon et al. [18]. Variables influencing decision regarding surgical approach and variables with potential influence on outcomes were assigned propensity scores using a bivariate logistic regression model. The final model included the following variables: age, Sex, BMI, ASA score, comorbidity, T stage and type of resection performed. We matched propensity scores 1:1 with the use of the nearest neighbour methods without replacement by using the closest calipers width to achieve the maximum number of cases without statistical differences in confounders variables. Survival analyses were conducted using the Kaplan–Meier method with log-rank test comparisons. Significance was defined as a* p* value less than 0.05. Statistical analysis was performed using the SPSS software 25.0 (SPSS, Inc., Chicago, IL).

## Results

Between September 2012 and March 2017, a total of 123 patients fulfilled the study criteria and were included in the analysis. Seventy-seven patients underwent an open gastrectomy while 46 patients were operated on by robotic approach.

### Baseline variables before matching

No significant differences were observed between groups in terms of sex, ASA score, BMI, comorbidities, Charlson Age Comorbidities Index (CACI) and tumor histotype. (Table 1). Significantly elderly patients were operated in the open group compared to robotic (80.6 ± 5.8 years vs. 77.5 ± 4.2 years; *p* = 0.003). Finally, in the unmatched cohorts, more patients underwent distal gastrectomy by robotic approach (58.4% vs. 73.9%, *p* = 0.124); whereas the majority of total gastrectomy were performed in the open group (27.3% vs. 19.6%, *p* = 0.124). This results from the different tumor location: RG group accounts more distal tumors (42.9% vs. 67.4%) while OG group had more proximal neoplasms (24.7% vs. 10.9%), (*p* = 0.029).

**Table 1:** Baseline characteristics before and after propensity score matching

	Before propensity score matching		*p*	After propensity score matching		*p*
	Open *n* = *77*	Robotic *n* = *46*		Open *n* = *43*	Robotic *n* = *43*	
Age (years, mean ± SD)	80.6 (± 5.8)	77.5 (± 4.2)	0.003	78.5 (± 5.3)	77.7 (± 4.2)	0.522
Gender F/M	32/45	25/21	0.169	22/21	23/20	0.829
BMI (mean, ± SD)	23.0 (± 5.8)	23.2 (± 5.0)	0.908	23.8 (± 4.9)	23.3 (± 5.1)	0.492
ASA (*n*, %)			0.552			0.787
1	1 (1.3%)	0 (0.0%)		0 (0.0%)	0 (0.0%)	
2	26 (33.8%)	16 (34.8%)		16 (37.2%)	13 (30.2%)	
3	42 (54.5%)	28 (60.9%)		25 (58.1%)	28 (65.1%)	
4	8 (10.4%)	2 (4.3%)		2 (4.7%)	2 (4.7%)	
Comorbidities (*n*, %)	72 (93.5%)	40 (87.0%)	0.365	41 (95.3%)	40 (93.0%)	1.000
CACI (median, range)	5 (3–9)	4 (3–11)	0.148	4 (3–8)	4 (3–11)	0.729
Histotype			0.551			0.663
Intestinal	39 (50,6%)	29 (63.0%)		23 (53.5%)	26 (60.5%)	
Diffuse	25 (32.5%)	11 (23.9%)		16 (37.2%)	11 (25.6%)	
Signet-ring cell	4 (5.2%)	2 (4.3%)		1 (2.3%)	2 (4.7%)	
Mucinous	9 (11.7%)	4 (8.7%)		3 (7.0%)	4 (9.3%)	
Tumor location			0.029			0.401
Subcardial	12 (15.6%)	4 (8.7%)		4 (9.3%)	4 (9.3%)	
Fundus	7 (9.1%)	1 (2.2%)		3 (7.0%)	1 (2.3%)	
Body	15 (19.5%)	8 (17.4%)		8 (18.6%)	8 (18.6%)	
Angulus	5 (6.5%)	0 (0.0%)		2 (4.7%)	0 (0.0%)	
Antrum	29 (37.7%)	31 (67.4%)		21 (41.8%)	28 (65.1%)	
Pylorus	4 (5.2%)	0 (0.0%)		2 (4.7%)	0 (0.0%)	
Gastric stump	5 (6.5%)	2(4.3%)		3 (7.0%)	2 (4.7%)	
Tumor size (cm, mean ± SD)	6.4 (± 4.1)	4.6 (± 2.5)	0.073	5.8 (± 3.8)	4.6 (± 2.5)	0.267

*BMI* body mass index, *ASA* American Society of Anesthesiology, *CACI* Charlson Age-Comorbidities Index

### Baseline variables and short-term outcomes after matching

After propensity score matching, 43 OG were compared to 43 RG. Distal gastrectomy was the most common type of operation performed in both groups (62.8% in OG vs. 72.1% in RG; *p* = 0.654).

The mean operative time was significantly lower in the OG: 189.3 ± 58.7 vs. 267.9 ± 88.0 min *p* < 0.001. Conversion during RG occurred in 6 (14%) patients: for uncontrolled bleeding (2 patients), for the severe adhesive syndrome (1 patient) and for the infiltration of the transverse mesocolon or anterior pancreatic capsule (3 patients). The blood loss did not differ between the two groups: 85.0 ± 27.6 vs. 86.7 ± 71.1 mL,* p* = 0.198. Postoperative outcomes are depicted in Table 2. Time to first flatus (*p* = 0.471) as well as time to first stool (*p* = 0.222) and time to soft oral intake (*p* = 0.380) are comparable between OG and RG. Postoperative pain was significantly lower in the RG (1.24 ± 0.7 vs. 0.95 ± 0.7, *p* = 0.042). Morbidity was similar between groups (*p* = 0.662) with most of the complications being minor according to Clavien-Dindo classification (25.6% in OG vs. 30.2% in RG *p* = 0.198). Furthermore, major complications mostly occurred after open surgery (16.3% vs. 6.9%, OR 2.592, 95% CI 0.623–10.785, *p* = 0.313). In particular, in the OG group three patients underwent reoperation: one patient for cholecystitis with pancreatitis, one for intestinal occlusion by ileal volvulus and one for biliary peritonitis. One patient underwent pleural drainage for pleural effusion, one patient to endoscopic dilatation of anastomotic stenosis and the last patient with major complication suffered from an anastomotic leak conservatively treated, but developed stroke and pleural effusion (Table 2).

**Table 2:** Operative and postoperative outcomes before and after propensity score matching

	Before propensity score matching		*p*	After propensity score matching		*p*
	Open *n* = *77*	Robotics *n* = *46*		Open *n* = *43*	Robotics *n* = *43*	
Type of gastrectomy (*n*, %)			0.124			0.654
Distal	45 (58.4%)	34 (73.9%)		27 (62.8%)	31 (72.1%)	
Total	21 (27.3%)	9 (19.6%)		12 (27.9%)	9 (20.9%)	
Degastro-gastrectomy	7 (9.1%)	3 (6.5%)		4 (9.3%)	3 (7.0%)	
Proximal	4 (5.2%)	0 (0.0%)		0 (0.0%)	0 (0.0%)	
Operative Time (min, mean ± SD)	188.2 (**± **66.5)	267.4 (**± **83.3)	< 0.001	189.3 (**± **58.7)	267.9 (**± **88.0)	< 0.001
Conversion (*n*, %)		8 (17.4%)			6 (14%)	
Blood loss (mean ± SD)	83.8 (**± **29.3)	85.9 (**± **68.7)	0.268	85.0 (**± **27.6)	86.7 (**± **71.1)	0.198
Pain after 6 h (VRS, mean ± SD)	1.29 (**± **0.9)	0.93 (**± **0.8)	0.029	1.24 (**± **0.7)	0.95 (**± **0.7)	0.042
Time to first flatus (days, mean ± SD)	4.3 (± 1.2)	4.4 (± 0.9)	1.000	4.5 (± 1.2)	4.4 (± 0.9)	0.471
Time to first stool (days, mean ± SD)	5.8 (± 2.0)	5.5 (± 0.9)	0.403	6.0 (± 2.3)	5.5 (± 0.9)	0.222
Time to oral intake (days, mean ± SD)	4.9 (± 1.8)	4.9 (± 0.8)	0.223	5.0 (± 2.0)	4.9 (± 0.8)	0.380
Hospital stay (days, median)	9 (6–27)	9 (7–90)	0.454	9 (6–25)	9 (7–90)	0.685
30 days morbidity (Clavien-Dindo I–IV) (*n*, %)	30/77 (39.0%)	18/46 (39.1%)	1.000	18/43 (41.9%)	16/43 (37.2%)	0.662
Clavien-Dindo I–II	20/77 (25.9%)	15/46 (32.6%)	0.431	11/43 (25.6%)	13/43 (30.2%)	0.806
Clavien-Dindo III–IV	10/77 (12.9%)	3/46 (6.5%)	0.367	7/43 (16.3%)	3/43 (6.9%)	0.313
Re-operation (*n*, %)	8/77 (10.4%)	2/46 (4.3%)	0.318	5/43 (11.6%)	2/43 (4.7%)	0.433
30 days mortality (*n*, %)	4/77 (5.2%)	0/46 (0.0%)	0.296	1/43 (2.3%)	0/43 (0.0%)	1.000

*VRS* Verbal Rating Scale

Concerning the RG group, two patients underwent reoperation: one for the leak of the duodenal stump and the other for a twisting of the gastro-jejunal anastomosis. The third patient with major complication underwent endoscopic dilatation of anastomotic stenosis.

Finally, length of hospital stay was comparable between groups (9 vs. 9 days; *p* = 0.685).

### Pathological and long-term outcomes

Pathological examination of the resected specimen showed comparable sizes of the tumor (5.8 vs 4.6 cm; *p* = 0.267), T stage (*p* = 0.575), N stage (*p* = 0.340) and M stage (*p* = 1.000) between groups (Table 3). Furthermore, overall R0 resection was achieved in 86.0% and 95.3%, of cases in OG and RG respectively (*p* = 0.181).

**Table 3:** Oncological outcomes

	Open *n* = *43*	Robotic *n* = *43*	*p*
T-stage (*n*, %)			0.575
pT1	5 (11.6%)	11 (25.6%)	
pT2	7 (16.3%)	7 (16.3%)	
pT3	13 (30.2%)	13 (30.2%)	
pT4	18 (41.8%)	12 (27.9%)	
N-stage (*n*, %)			0.340
pN0	14 (32.6%)	23 (53.5%)	
pN1	7 (16.3%)	5 (11.6%)	
pN2	6 (14.0%)	6 (14.0%)	
pN3	16 (37.2%)	9 (20.9%)	
M-stage (*n*, %)			1.000
pM0	38 (88.4%)	38 (88.4%)	
pM1	5 (11.6%)	5 (11.6%)	
R0 resection (*n*, %)	37 (86.0%)	41 (95.3%)	0.181
Retrieved nodes (mean ± SD)	22.5 (± 12.8)	22.1 (± 8.4)	0.856
Positive nodes (mean ± SD)	7.4 (± 11.1)	4.0 (± 6.9)	0.053
Node ratio (mean ± SD)	0.3 (± 0.3)	0.2 (± 0.3)	0.043
TNM stage (*n*, %)			0.627
IA	4 (9.3%)	8 (18.6%)	
IB	3 (7.0%)	7 (15.2%)	
IIA	9 (20.9%)	8 (18.6%)	
IIB	4 (9.3%)	4 (9.3%)	
IIIA	5 (11.6%)	3 (7.0%)	
IIIB	3 (7.0%)	4 (9.3%)	
IIIC	10 (23.3%)	4 (9.3%)	
IV	5 (11.6%)	5 (11.6%)	

Finally, the number or retrieved lymph nodes (22.5 vs 22.1, *p* = 0.856) was comparable between groups, but the open group had more metastatic lymph nodes (7.4% vs. 4.0%, *p* = 0.053) and a higher lymph node ratio (0.3 vs. 0.2, *p* = 0.043).

**Fig. 1:**
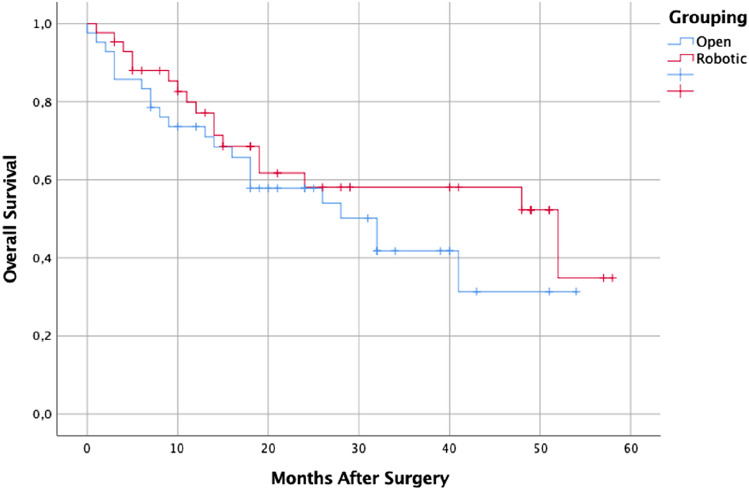
Patient’s Overall Survival According to Surgical Approach

**Fig. 2:**
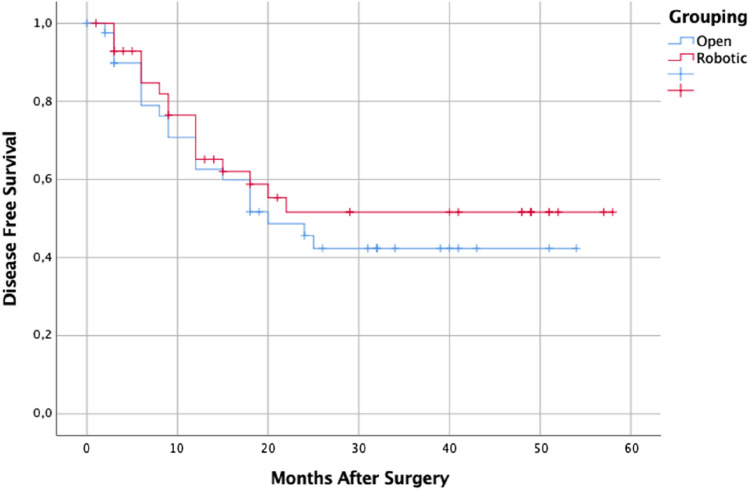
Patient’s Disease Free Survival According to Surgical Approach

After a median follow-up of 32.0 (16.8–47.2) months for OG and 52.0 (15.3–88.7) months for RG, the 1–3-5 years OS rate was 73.7%, 41.8% and 31.3% for open and 82.7%, 52.3% and 34.9% for robotic (*p* = 0.263; Fig. 1); whereas 1–3-5 years DFS rate was 62.6%, 42.4% and 42.4% for open and 65.1%, 51.6% and 51.6% (*p* = 0.474; Fig. 2).

## Discussion

The elderly population is steeply increasing worldwide but there is no agreement on a definition of elderly. Many studies define as “elderly” only patients older than 75 years [19, 20]. Nowadays, the WHO still considers as elderly those individuals of 60 years or over; however, most scientific societies define patients as elderly if their age is 65 year or more.

Elderly patients have an increased operative risk and high postoperative morbidity and mortality rate because of associated diseases such as hepatic, cardiovascular, and pulmonary diseases [21]. Thus, elderly patients are often unable to bear neoadjuvant or adjuvant chemotherapy [22].

Despite that, nowadays it is not justified to deny surgical procedures to elderly patients based only on age. Elderly patients who survive the first year after surgery have been shown to have the same cancer-related survival as younger patients [23].

However, as major abdominal surgery in elderly and frail patients is related to a higher risk of morbidity and mortality, they can benefit from an integrated, team-based approach. This should include geriatricians, anesthesiologists, oncologists and surgeons working together to optimize drug management eventually reducing general morbidity and acute geriatric events as well as other complications and the total length of hospitalization [24, 25].

The advantages of minimally invasive surgery (MIS) characterized by small incisions has provided many benefits over open surgery including less intraoperative blood loss, faster postoperative bowel function, shorter hospital stay, less postoperative pain, fewer wound infections, and a lower incidence of postoperative pneumonia and incidence of postoperative heart complications. All those benefits were widely demonstrated in the Eastern multicentric randomized control trials of high-volume centers for both early and locally advanced gastric cancer; for that reason, we recently investigated our results about locally advanced gastric cancer in middle-low volume centers in a western setting [26–28].

Robotic surgery must be considered as the natural evolution of conventional MIS laparoscopy, consisting of a computerized interface to facilitate intuitive movements (EndoWristTM System) similar to conventional open surgery.

Focusing on gastric surgery, the introduction of robotic-assisted technique in the early 2000s allowed to overcome some technical limitations of laparoscopy, especially during the reconstructive time and the execution of D2 lymphadenectomy [7–9]. Robotic technology enables accurate dissection of lymphocellular tissue, reducing the risk of bleeding. The advantages are particularly evident when the lymph node dissection must be conducted circumferentially around the vessels [29, 30]. In fact, in some series the average number of lymph nodes removed with the robotic technique was superior to laparoscopy [13, 31].

Since that time, several studies have demonstrated the safety and efficacy of robot-assisted gastrectomy in the treatment of gastric adenocarcinoma [10, 32, 33].

At present, there is no evidence that robotic surgery should be considered better than minimally invasive conventional surgery, even because overall operating times and costs are still higher than laparoscopy [9, 12, 31].

On the other hand, robotic surgery increases MIS access to patients by reducing the overall conversion rate and learning curve compared to conventional laparoscopy [34, 35]. Furthermore, the dual console platform allows a step by step teaching in the setting of a residency program on robotic surgery.

The present study, focusing on the safety and feasibility of robotic gastric surgery, highlights that this surgical approach could lead to possible benefits in the elderly population.

Indeed, the reduced odds ratio of major postoperative complications associated with a significant lower postoperative pain seems to be an important advantage for such a frail cohort of patients.

In fact, it is well known that the reduction in postoperative pain is very important for the patient and for their total experience of the surgical procedure. More important still is the association between postoperative pain and immune deficiency, wound healing and the occurrence of chronic pain [36, 37]. The reduction of postoperative pain experienced by the patient improves mobilisation and ultimately reduces adverse postoperative outcome [38].

Moreover, the reduced consumption of analgesics could lead to a lower risk of pharmacological interactions in patients with severe comorbidities and multidrug therapies.

Unlike what has been reported, no differences were found in the current series between the two groups regarding the hospital stay. This finding is not surprising when Enhanced Recovery After Surgery (ERAS) Protocols are not applied. In fact, the postoperative protocol was the same for all patients, regardless of the surgical approach.

Concerning the oncological safety of the robotic technique, in the current series all patients underwent a D2 lymphadenectomy with preservation of the pancreas and spleen. The average number of lymph nodes removed was above the minimum recommended by Japanese and Western guidelines [39, 40].

Finally, regarding the long-term results, the majority of the literature compared robot-assisted surgery and laparoscopy, without differences in terms of disease-free and overall survival [41–43]. In our series, as shown by other studies comparing robotic and open gastrectomy, no significant differences were observed in terms of OS and DFS between the two surgical approaches [44]. Probably the better survival curves of the RG group were mainly due to the higher amount of metastatic lymph nodes in the OG group.

The results of an ongoing multicentric observational trial comparing robotic, laparoscopic and open surgery for gastric cancer (IMIGASTRIC) will add some evidence to the current debate [45].

The most important limitations of the present study are the retrospective fashion of the study and the sample size. As stated above, the incidence of gastric cancer in western countries is limited; thus, it is the reason for our sample size. However, it is important to consider that the propensity score model allowed us to compare two similar groups.

In conclusion, this study confirms that robotic surgery for gastric cancer is safe and feasible, showing similar short and long-term outcomes compared to the open technique. Despite the higher operative time and costs, robotic technology appears promising in reducing the postoperative pain and the rate of major complications. The duration of surgery and pneumoperitoneum seems to not have a negative effect on elderly patients which might benefit from a probably reduced rate of postoperative incisional hernia.
